# Assessing the impact of jail-initiated medication for opioid use disorder: A multisite analysis of the SOMATICS collaborative

**DOI:** 10.1371/journal.pone.0305165

**Published:** 2024-06-17

**Authors:** Joshua D. Lee, Keith Goldfeld, Robert P. Schwartz, Ryan McDonald, Yifan Xu, Redonna Chandler, Kevin Hallgren, Sharon M. Kelly, Shannon Gwinn Mitchell, Anjalee Sharma, David Farabee

**Affiliations:** 1 Department of Population Health, NYU Grossman School of Medicine, New York City, NY, United States of America; 2 Friends Research Institute, Baltimore, MD, United States of America; 3 National Institute on Drug Abuse, Bethesda, MD, United States of America; 4 Department of Psychiatry and Behavioral Sciences, University of Washington, Seattle, WA, United States of America; 5 Johns Hopkins School of Medicine, Baltimore, MD, United States of America; University of Miami, UNITED STATES

## Abstract

The objective of this study was to estimate the associations of jail-initiated medication for opioid use disorder (MOUD) and patient navigation (PN) with opioid use disorder (OUD) at 6 months post-release. Three randomized trials (combined N = 330) were combined to assess whether MOUD (extended-release naltrexone or interim methadone) initiated prior to release from jail with or without PN would reduce the likelihood of a DSM-5 diagnosis of OUD 6 months post-release relative to enhanced treatment-as-usual (ETAU). Across the three studies, assignment to MOUD compared to ETAU was not associated with an OUD diagnosis at 6 months post-release (69% vs. 75%, respectively, OR = 0.67, 95% CI: 0.42 to 1.20). Similarly, PN compared to MOUD without PN was not associated with an OUD diagnosis (63% vs 77%, respectively, OR = 0.61, 95% CI: 0.27 to 1.53). Results underscore the need to further optimize the effectiveness of MOUD for patients initiating treatment in jail, beginning with an emphasis on post-release treatment adherence.

## Introduction

The increasing incidence of opioid-involved overdose deaths in the U.S. pervades many sectors of the population, but the risk of a fatal overdose is especially high among those leaving jail or prison [1]. Reduced tolerance for opioids due to abstinence or reduced use while incarcerated substantially increases the risk of overdose if the released individual resumes illicit opioid use in the community. In fact, formerly incarcerated adults have more than a four-fold risk of dying from drug overdose than their demographically-similar counterparts who have not been incarcerated [2].

Despite the high prevalence of opioid use disorder (OUD) and overdose risk, jail-initiated medication for opioid use disorder (MOUD) remains scarce. One national survey found on admission 14.5% of jail residents in June 2019 screened positive for OUD, 4.8% received opioid withdrawal treatment, and only 0.9% of jail residents received MOUD [3]. In the U.S. there are currently two medications approved by the FDA for treating OUD (methadone and buprenorphine) and one approved for the prevention of OUD relapse (extended-release naltrexone), with only 1 in 5 jails making MOUD available to any resident with OUD [4]. Even among women who are pregnant at the time of incarceration, only 60% of jails continue to provide methadone or buprenorphine to those in treatment at the time of arrest, and only about one third *initiate* one of these medications while in custody [5]. From a public health perspective, the limited availability of MOUD in jails may be a critical oversight, as local jails typically admit more than 10 million adults per year (though the most recent data from 2020 showed a decline to 8.7 million during the pandemic) [6].

Although there is strong established empirical support for MOUD in community settings, its effectiveness for incarcerated patients is less clear. A number of corrections-based experimental evaluations of MOUD in US settings exist [7–12] or are underway [13], but variations in study correctional settings (prison vs. jails), designs, and measures hamper efforts to generate stable estimates of the effects of jail-initiated MOUD on post-release opioid use, OUD, and overdose. After systematically reviewing 20 CLS-based MOUD studies, Strange and colleagues found that provision of MOUD in these settings was associated with a significantly reduced the risk of non-fatal opioid overdose following release but not with reduced risk of fatal overdose or recidivism. The authors suggested the null effect on fatal overdoses could be due to difficulty in accessing mortality records [14]. and highlighted the need for more rigorous research in this area, including the use of experimental designs.

The Studies on Medications for Addiction Treatment in Correctional Settings (SOMATICS) collaborative was established to address some of these limitations. The findings of its individual studies have been reported elsewhere [7, 11, 12]. Using harmonized data, the three studies from the collaborative were designed to examine whether MOUD initiated prior to release from jail with or without patient navigation (PN) would reduce the likelihood of an OUD diagnosis at 6 months following release.

## Materials and methods

The SOMATICS research collaborative included three research centers (RCs) each conducting an individual randomized trial while sharing the Enhanced Treatment as Usual (ETAU) arm and collecting data using several core assessments common across all sites. ETAU refers to standard treatment at the facilities without planned MOUD initiation to continue post-release, enhanced by the provision of overdose education and community-based treatment referrals. The SOMATICS RCs conducted three open-label randomized effectiveness trials among male and female adults with OUD comparing ETAU to the initiation of medication—extended-release naltrexone (XR-NTX) in sentenced jail residents–in Sites 1 (in New York City) and 2 (in Albuquerque, NM) and methadone in pre-trial jail detainees in Site 3 (in Baltimore, MD). Additionally, it tested the benefit of offering patient navigation (PN) to patients assigned to an MOUD condition vs. MOUD alone in Sites 2 and 3 (Table 1). Recruitment began at each of the three RC sites at different times. The first participant was enrolled on July 10, 2014 and the final participant was enrolled on December 11, 2018. The combined data from these trials allowed us to estimate the benefit of medications with and without patient navigation for individuals transitioning from jail to the community under a variety of real-world conditions.

**Table 1:** SOMATICS collaborative study arms by site.

Site	Medication	Medication and PN	ETAU	Totals
**Site 1** **New York City**	XR-NTX n = 58	-	n = 52	**110**
**Site 2** **Albuquerque**	XR-NTX n = 29	n = 21	n = 19	**69**
**Site 3** **Baltimore**	IM n = 46	n = 54	n- = 51	**151**
**Totals**	**133**	**75**	**122**	

IM = Interim Methadone; PN = Patient Navigation

ETAU = Enhanced Treatment-as-usual

Note: Randomization allocation for Site 1 was 1:1. For Sites 2 & 3, the allocation was 1:1:1

### Research questions and hypotheses

The SOMATICS collaborative’s primary research question addressed whether MOUD (XR-NTX or methadone) initiated prior to release from jail, alone or in combination with patient navigation (PN), would reduce the likelihood of a DSM-5 OUD diagnosis at 6 months following release. We hypothesized that medication initiated in jail would have superior outcomes compared to ETAU. In addition, we hypothesized that the groups assigned to the combination of medication and PN would evince superior outcomes compared to medication alone.

### Study organization and sites

Three independent RCs implemented different protocols under an NIH collaborative agreement. Site 1: New York University Grossman School of Medicine and Bellevue Hospital Center working in New York City Department of Corrections facilities on Rikers Island with sentenced residents (New York, NY); Site 2: University of California Los Angeles (UCLA) and the University of New Mexico in collaboration with Bernalillo County Metropolitan Detention Center with sentenced residents (Albuquerque, NM); Site 3: Friends Research Institute in collaboration with Maryland Division of Pretrial and Detention Services with pre-trial detainees (Baltimore, MD). For detailed descriptions of each site/study’s methodology, including inclusion criteria and recruitment procedures, see published protocols [15–17]. Study-specific inclusion/exclusion criteria are also provided in the supplemental material for this paper (S1 Appendix).

### Regulatory affairs and data and safety monitoring

Each study participant provided written, informed consent prior to enrollment, witnessed by the local research associate. Participants were informed their participation was voluntary and that they could leave the study at any time without consequences from the study team or corrections staff. In compliance with requirements for research with “prisoners”, all participants had the opportunity to receive additional services through the study that were not available to non-participants. At a minimum, research staff provided study participants with overdose educational information and a list of community treatment resources, which were not otherwise systematically available at the facilities at that time. Detainees who were not enrolled in methadone maintenance in the community at the time of arrest in Baltimore were not able to start methadone maintenance during detention. Similarly, it was not possible to start extended-release naltrexone at the New York and NM sites. Those opting not to participate could access other forms of treatment offered by the jail-based healthcare providers.

All three SOMATICS studies were approved by site specific Institutional Review Boards (IRB) located at NYU, UCLA, and FRI which transferred IRB oversight during the study to the Western IRB. In addition, the U.S. Office of Human Research Protections approved study protocols and agreed with IRB determinations that the research was being conducted with prisoners in accordance with 44 CFR 46.3069(a)(2)(iv). Each study was registered with ClinicalTrials.gov (NCT01999946, NCT02110264, NCT02334215). Federal Certificates of Confidentiality were obtained for each study to ensure protection of research participant data. Lastly, a single Data and Safety Monitoring Board located at UCLA’s Integrated Substance Abuse Programs monitored the studies.

### Participants

**Fig 1:**
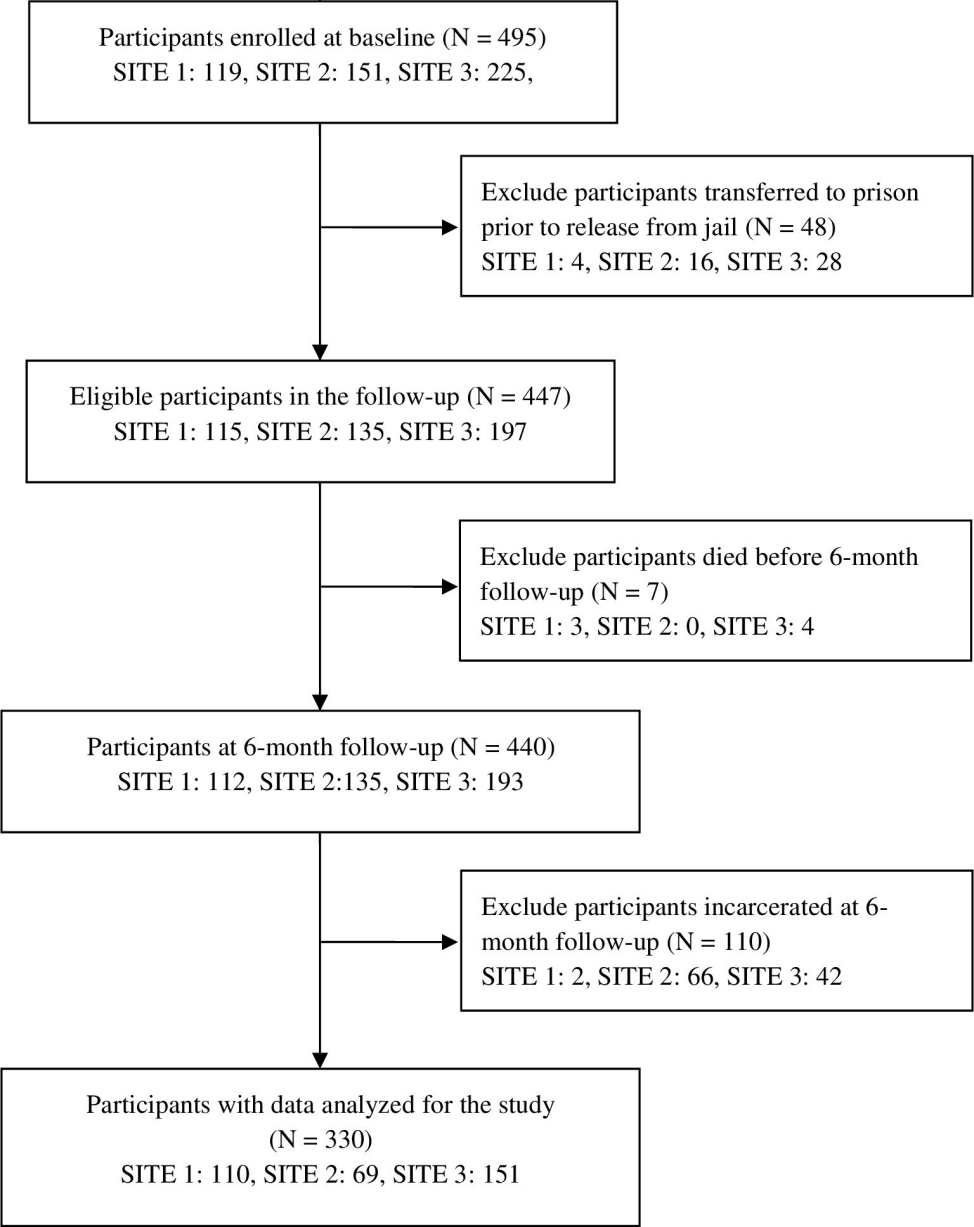
SOMATICS study consort diagram.

Participants in Sites 1 and 2 were recruited from among adults serving short sentences in jail. Site 3 participants were recruited from adults recently entering pre-trial detention. Following informed consent, participants were randomly at each site to study condition on a 1:1 basis (Fig 1). For the present analysis, data were excluded for two reasons unique to conducting research in jails. First, data were excluded for 28 pre-trial participants in Site 3 who were sentenced and transferred to prison and 20 participants in Sites 1 and 2 (combined) who were transferred directly to prison from jail because their medications were discontinued and their release was delayed. Second, 110 participants who were due to be interviewed for their 6-month follow-up while incarcerated following re-arrest were not included (Fig 1) because not all study sites were able to access participants who had become re-incarcerated.

### Assessments

Across the three sites, participants were administered a modified World Mental Health-Composite International Diagnostic Interview (WMH-CIDI) [18] at baseline (regarding the past 12 months) and at 6 months (regarding the past month) following release from the index incarceration to determine if criteria were met for a current (i.e., not “in remission”) DSM-5 OUD diagnosis. Missing assessments for individuals who were alive and in the community (not incarcerated) at the time of their 6-month assessment were considered positive for having met current DSM-5 criteria for OUD.

### Treatment conditions

For Sites 1 and 2, the XR-NTX condition began with a first dose prior to release and participants were offered XR-NTX for up to 6 months via monthly injections in the community following release. For Site 3, methadone administration began within a few days following arrest and consisted of methadone maintenance without counseling (interim methadone) and transfer to one of the participating community opioid treatment programs for continued methadone maintenance with counseling [11]. No additional counseling was offered to any of the conditions during incarceration, although participants were free to attend any counseling offered at their facility. Patient navigation at Sites 2 and 3 used the same PN manual and began with one session in jail and the availability of three months of navigation following release. The ETAU condition provided overdose prevention information and community treatment referrals at all sites and brief opioid withdrawal management with methadone at site 3.

### Statistical analyses

Sample characteristics were summarized by individual study, by treatment across studies, and for the combined sample, with means and standard deviations for continuous variables and frequencies and percentages for categorical variables. The primary outcome analysis comparing any MOUD with ETAU was conducted in two steps. In the first step, we used a logistic model to estimate the study-specific treatment associations (the log-odds ratios ${\hat{\theta }}_{j},j\in \{1,2,3\}$) after adjusting for selected participant-level characteristics including age, sex, race, housing status (housed vs. unhoused), and cocaine/amphetamine use prior to enrollment. We then estimated an overall, pooled treatment association (log-odds ratio ${\hat{\theta }}_{p}$) by weighting the study-specific treatment associations by the inverse variance of the estimates from step one. We conducted a hypothesis test of the pooled log-odds ratio $\left(H_{0}:\theta_{p}\leq 0\;vs\;H_{1}:\theta_{p}>0\right)$ using a permutation test and estimated a 95% confidence interval using bootstrap methods. We used the same approach to compare medication and PN with medication alone in Sites 1 and 2; the only difference is that since only two studies were used in this analysis, ${\hat{\theta }}_{p}$ was based on the weighted average of ${\hat{\theta }}_{j},j\in \{1,2\}$. As a sensitivity analysis, we developed Bayesian models to provide an alternative estimate of the pooled treatment association, and we reported 95% credible intervals of the study-specific and pooled log-odds ratios based on the estimated posterior distributions.

## Results and discussion

### Study participants

Table 2 shows the baseline characteristics for 330 participants among all three studies and for each of the treatment arms (ETAU vs. MOUD) of the combined SOMATICS study sample. Approximately one in five (21%) were female, and the mean age was 39 (SD = 11). With regard to race/ethnicity, 49% of the sample identified as Black, 41% as White, and 10% as members of other racial groups. Twenty-nine percent of the participants identified their ethnicity as Hispanic. The majority (63%) reported having at least a high-school degree, and approximately one-third (32%) described themselves as being homeless at the time of incarceration. Thirty percent of the sample reported being positive for Hepatitis C.

**Table 2:** Baseline characteristics of the participants in SOMATICS by treatment condition.

	ETAU only (N = 122)	Any treatment (N = 208)	Overall (N = 330)
**Assignment**			
XR-NTX	0 (0.0%)	87 (41.8%)	87 (26.4%)
ETAU	122 (100.0%)	0 (0.0%)	122 (37.0%)
IM	0 (0.0%)	46 (22.1%)	46 (13.9%)
IM+PN	0 (0.0%)	54 (26.0%)	54 (16.4%)
XR-NTX+PN	0 (0.0%)	21 (10.1%)	21 (6.4%)
**Site**			
Site 1	52 (42.6%)	58 (27.9%)	110 (33.3%)
Site 2	19 (15.6%)	50 (24.0%)	69 (20.9%)
Site 3	51 (41.8%)	100 (48.1%)	151 (45.8%)
**Age, mean (SD)**	39.8 (11.0)	38.9 (10.8)	39.2 (10.9)
**Race**			
Black or African	59 (48.4%)	103 (49.5%)	162 (49.1%)
White	46 (37.7%)	89 (42.8%)	135 (40.9%)
Other	17 (13.9%)	16 (7.7%)	33 (10.0%)
**Hispanic**	41 (33.6%)	56 (26.9%)	97 (29.4%)
**Female**	28 (23.0%)	42 (20.2%)	70 (21.2%)
**High School or higher**	82 (67.2%)	126 (60.6%)	208 (63.0%)
**Single or not partnered**	78 (63.9%)	140 (67.3%)	218 (66.1%)
**Not working**	80 (65.6%)	102 (49.0%)	182 (55.2%)
**Unhoused**	35 (28.9%)	70 (33.7%)	105 (31.9%)
**On parole/probation**	41 (33.6%)	81 (39.3%)	122 (37.2%)
HIV positive	7 (6.3%)	13 (6.9%)	20 (6.7%)
Hep C positive	34 (34.0%)	49 (27.8%)	83 (30.1%)
Injected drugs in past 6 months	62 (50.8%)	105 (50.5%)	167 (50.6%)
Heterosexual	110 (90.2%)	197 (94.7%)	307 (93.0%)
Days used cocaine in the past 30 days, mean (SD)	11.6 (13.4)	9.4 (12.6)	10.2 (12.9)
Days used heroin in the past 30 days, mean (SD)	27.2 (6.6)	26.3 (7.8)	26.6 (7.4)
Days used amphetamines in the past 30 days, mean (SD)	4.1 (10.3)	4.7 (10.8)	4.5 (10.6)
Days used any stimulants in the past 30 days, mean (SD)	15.7 (13.7)	14.0 (13.6)	14.6 (13.7)

### Primary outcome: OUD diagnoses at 6 months

**Fig 2:**
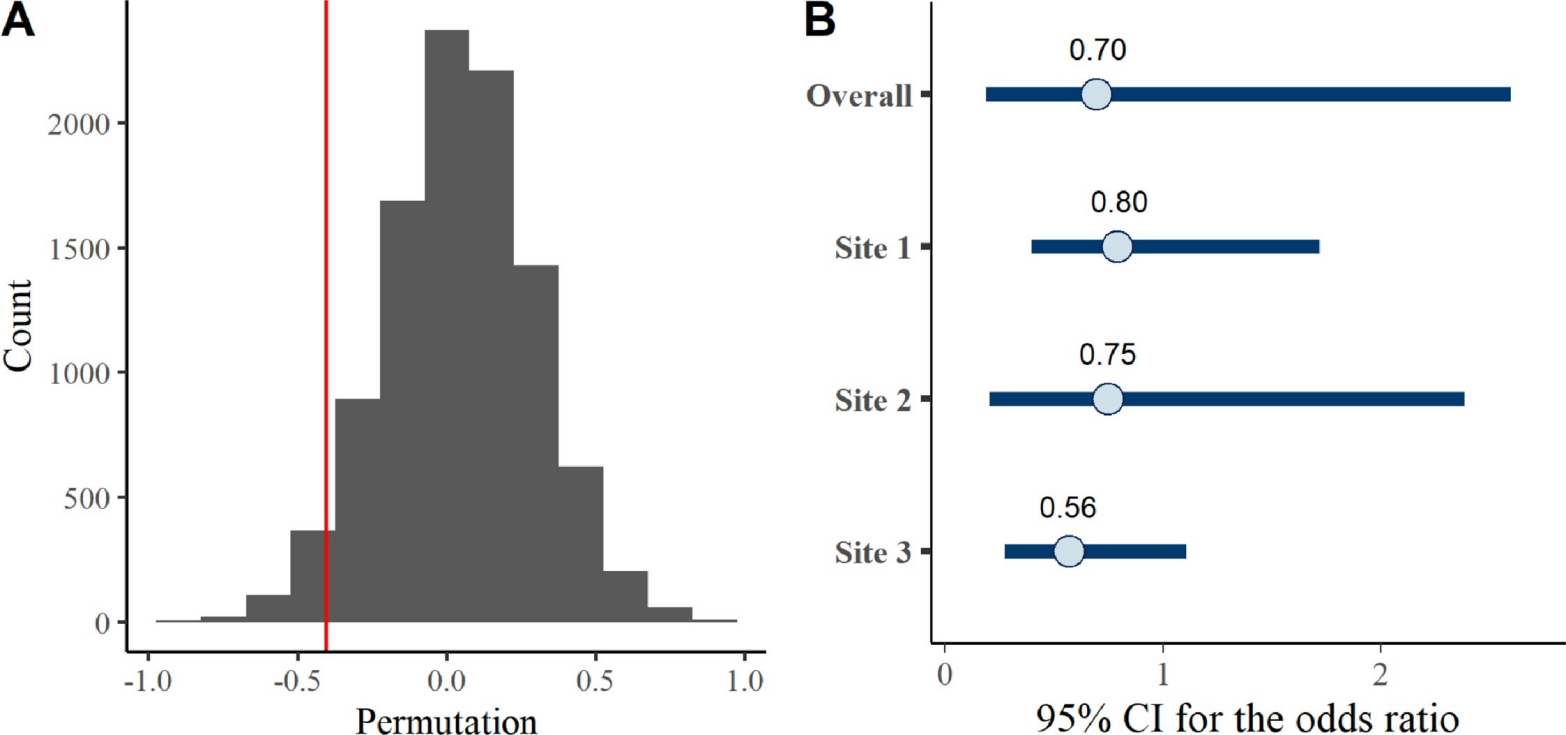
Permutation test of *log*(*θ*_*p*_) for MOUD in the 3-site model.

Across the three studies, 75% of the ETAU group had a current OUD diagnosis at 6 months following release compared with 69% of the participants who were assigned to receive any MOUD. After adjusting for participant characteristics, the pooled odds ratio (*θ*_*p*_) comparing OUD diagnosis in the treatment group with the ETAU group across all three studies was 0.67. The two-tailed p-value based on the permutation test was 0.11 (Fig 2), and the 95% confidence interval estimated using bootstrap sampling was 0.42 to 1.20. The pooled odds ratio using Bayesian approach was 0.70, and the 95% credible interval (Fig 2) was 0.19 to 2.59, which was consistent with the primary analysis.

**Fig 3:**
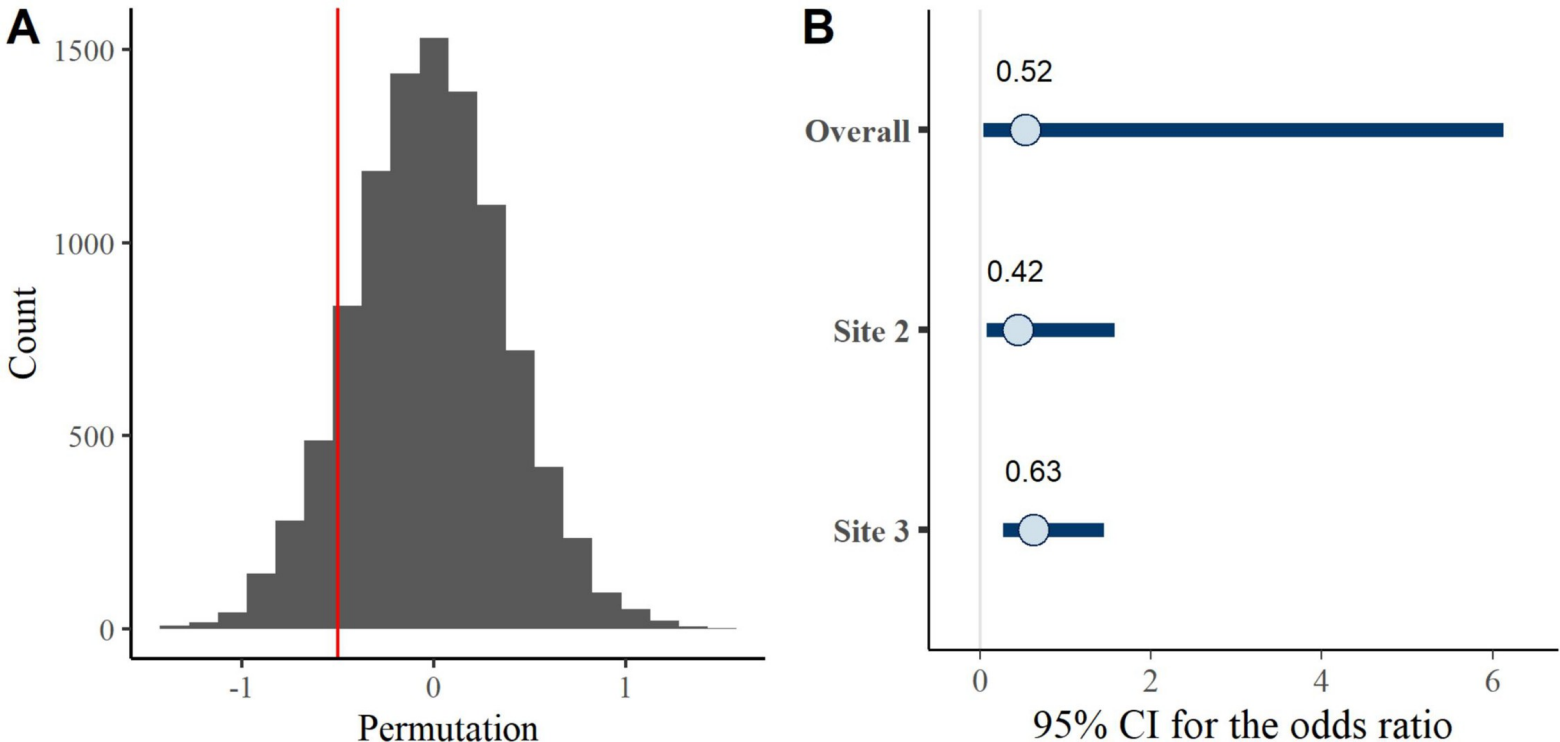
95% Bayesian credible interval of patient navigation effect (odds ratio) in the 2-site model.

With regard to PN, across both studies employing this approach, 77% of the medication-only participants met the criteria for OUD diagnosis at 6 months following release, while 63% of the medication plus navigation met OUD diagnostic criteria. The estimated odds ratio comparing medication plus navigation with medication only was 0.61 (permutation p-value 0.20 shown in Fig 3 –Panel A, 95% CI: 0.27, 1.53). The pooled odds ratio using Bayesian approach was 0.52, and the 95% credible interval (shown in Fig 3 –Panel B) was 0.04 to 6.13, also consistent with the primary analysis.

## Conclusions

In this study, data from three jail-based, randomized controlled trials were combined to assess the associations of pre-release MOUD initiation (methadone and extended-release naltrexone), alone or in combination with patient navigation (PN), with the likelihood of having a DSM-5 OUD diagnosis 6 months following release. Assignment to MOUD compared to ETAU was not associated with meeting criteria for OUD 6 months post-release. Likewise, assignment to MOUD with vs. without PN was not associated with this outcome.

The results of this study highlight the need to learn more about optimizing the effectiveness of these otherwise efficacious medications in community settings for patients initiating treatment in jail. One obvious target should be medication adherence. Only 24% and 8% of XR-NTX participants at Sites 1 and 2, respectively, received six months of scheduled post-release medication treatment [7, 12]. In the Site 3 methadone study, the majority of the participants were no longer enrolled in any form of OUD treatment 12 months following release. [11] Although patient navigation was included as a strategy to improve entry and retention in community-based treatment, it did not have a significant impact on 6-month OUD diagnosis in the SOMATICS cooperative. Perhaps this is not surprising, given that the patient-navigation intervention was not available after the third month following release. In subsequent qualitative research, SOMATICS participants offered some guidance as to how PN might be made more effective, such as offering longer periods of involvement and/or combining PN with contingency management (CM) [19]. A recent review of retention strategies for MOUD indicated an effect of CM for antagonist but not agonist treatments [20]. A recent qualitative study of patient navigators working with opioid users leaving prison illuminated the complex and challenging role of patient navigators when dealing with this high-risk, high-need population. In particular, the researchers found that navigators can be highly effective advocates in providing patients with psychosocial support and connecting them to community-based care, but less effective in assisting participants overcome larger social and structural barriers to recovery [21].

Another potential adaptation to studies of MOUD would be to examine various types of counseling associated with the delivery of medication in jail, although this would be limited in pre-trial settings which can be of extremely short duration.Such approaches could be delivered by behavioral health staff in-person or remotely or could include web or app-based interventions. There is also strong evidence for quarterly Recovery Management Checkups in the community, which have been shown to improve SUD treatment retention and drug-use outcomes [22].

The site-specific analysis reported elsewhere of the SOMATICS methadone study found that starting methadone maintenance in jail was associated with significantly higher rates of being in OUD treatment at 1-month post-release compared to ETAU, although there were no significant differences over the 12 month follow-up period [11]. The relatively low median methadone doses at release and 1, 3, and 6 months post-release (40 mg, 55 mg, 60 mg and 70 mg, respectively) may have contributed to the null finding in the present report.

The design of the SOMATICS collaborative offered a number of important methodological strengths, including randomized designs with harmonized assessments and observations periods. The combination of these demographically diverse study samples enhanced statistical power and generalizability, offering an advantage over many single-site studies. Potential limitations include the reliance on the self-reported primary outcome of meeting DSM-5 criteria for OUD with a low threshold of needing to meet only two of its 11 criteria. DSM OUD criteria has been infrequently used as an outcome in studies of MOUD, however, it has been recommended as key outcome in measurement-based care [23]. Additional limitations include the imputation of missing responses as positive, the brief (only 3 months) duration post-release PN services of the patient navigation intervention, and the omission of buprenorphine in the MOUD condition. There is evidence that self-reported drug use among CLS-involved populations is more valid among those who have participated in SUD treatment than those who have not [24]. The decision to impute missing responses as positive undoubtedly produced higher estimates of OUD prevalence than would have been obtained by simply dropping these participants from the analysis, as demonstrated by Wu et al., [25] but this was an a priori decision by the investigators to ensure the most conservative estimates of effectiveness (Indeed, a supplemental analysis without imputing missing DSM-5 OUD diagnoses as “positive” showed a relatively strong treatment effect, but this effect was generated by a non-randomized study design where missingness varied by condition.) The omission of buprenorphine as part of the MOUD condition primarily reflected usual practices (favoring methadone or no MOUD) at the three study sites during 2012–2017, as well as the relative lack of effectiveness data for XR-NTX prompting further study. Research on post-incarceration retention and outcomes for extended-release buprenorphine in comparison to sublingual buprenorphine and XR-NTX is currently being conducted by the SOMATICS investigators and colleagues [13]. Finally, the methadone study was conducted in a pre-trial population compared with a short-sentenced jail population in the other two studies, which may have led to unknown differences among the sites.

The results from this study suggest that initiating MOUD did not significantly reduce the occurrence of OUD 6 months following release compared to enhanced treatment as usual. Similarly, patient navigation in combination with MOUD, implemented by two of the study sites, did not appear to significantly improve this outcome. These results are consistent with the findings of the three SOMATICS studies that have presented site-specific outcomes [7, 11, 12]. In all three studies, there was a drop-off in the rates of receiving MOUD in the community following release, and there was entry into MOUD treatment in the ETAU conditions post-release. Neither of the medication+PN studies found a significant effect of medication or medication and PN compared to ETAU on treatment retention or opioid-use outcomes over 12 months post-release. Identifying effective strategies that will improve post-release MOUD adherence for patients who initiate treatment in jail will be critical in further reducing post-release relapse and overdose risk among the CLS population.

## Supporting information

S1 Appendix Inclusion/exclusion criteria. (DOCX)
