# Supplementary material for: Assessing the impact of jail-initiated medication for opioid use disorder: A multisite analysis of the SOMATICS collaborative
Source: PLoS One. 2024 Jun 17;19(6):e0305165. doi: 10.1371/journal.pone.0305165 (PMC11182542; doi:10.1371/journal.pone.0305165)
Supplement: S1 Appendix — (DOCX) [file pone.0305165.s001.docx]

**Appendix 1. Inclusion/Exclusion Criteria**

**Site 1:**

Inclusion criteria

1) Adults >18yo incarcerated in NYC jails with known release dates;

2) DSM-V criteria for current opioid use disorder (DSM-IV opioid dependence);

3) Not currently in or planning to pursue agonist (methadone, buprenorphine) treatment at release;

4) Currently opioid free by history (‘detoxed’) and with a negative urine for all opioids;

5) General good health as determined by medical evaluation;

Exclusion criteria:

1) Pregnancy, lactation, or planning conception;

2) Active medical illness (i.e., severe liver disease, congestive heart failure) precluding safe participation;

3) Untreated or poorly controlled psychiatric disorder precluding safe participation;

4) History of allergic reaction to naltrexone;

5) Current chronic pain condition treated with opioids.

**Site 2:**

Inclusion criteria

1) Be at least 18 years of age or older;

2) Meet criteria for DSM-5 opioid use disorders;

3) Be detained for at least 48 hours;

4) Have an expected release date within one year;

5) Plan to reside in area after release;

6) Have at least one instance of relapse to opioid use after a period of abstinence;

Exclusion criteria

1) Have a medical (e.g., liver failure, congestive heart failure) or psychiatric condition (e.g., suicidal ideation; psychosis) that would make participation unsafe in the judgment of the medical staff or the PI;

2) Have chronic pain and are currently or have plans to undergo pain treatment/therapy;

3) Have known sensitivity to naltrexone or naloxone;

4) Have participated in an investigational drug study within the past 30 days prior to screening;

5) Be a nursing or pregnant female, or not agree to use a medically acceptable form of birth control such as oral contraceptives, barrier (diaphragm or condom), levonorgestrel implant, intra-uterine progesterone contraceptives system, medroxyprogesterone acetate contraceptive injection, or complete abstinence; Females who become pregnant during the course of the study will be withdrawn from the study and, if requested, will be provided with referrals for drug treatment and/or medical care;

6) Have any pending legal action that could prohibit continued participation for the 24-week intervention period of the study, such as legal proceedings that could possibly result in incarceration;

7) Have a current pattern of alcohol, benzodiazepine, or other depressant or sedative hypnotic use, as determined by the study physician, which would preclude safe participation in the study;

**Site 3:**

Inclusion criteria:

1) Meets DSM-5 criteria for opioid use disorder;

2) Detained for at least 48 hours (because those detainees who are released quickly are most often released within 36 hours and hence would not have time to receive services in the study);

3) receiving opioid withdrawal treatment (as-usual) through the Detention Center’s medical providers;

4) Able and willing to provide informed consent in English;

5) Detained for a charge that, if found guilty, will likely result in a sentence of less than 1 year;

6) plan to reside in Baltimore upon release;

7) 18 years of age and older;

Exclusion criteria:

1) Enrolled in MM or buprenorphine treatment in the community at the time of arrest;

2) Having a medical (liver failure, congestive heart failure) or psychiatric condition (e.g., suicidal ideation, psychosis) that would make participation unsafe in the judgment of the medical staff or the PI;

3) Pregnancy;

4) Allergy to methadone; and,

5) Requiring treatment for alcohol or sedative hypnotic withdrawal (i.e., in response to moderate or severe withdrawal).
